# Sialylation of the prion protein glycans controls prion replication rate and glycoform ratio

**DOI:** 10.1038/srep16912

**Published:** 2015-11-18

**Authors:** Elizaveta Katorcha, Natallia Makarava, Regina Savtchenko, Ilia V. Baskakov

**Affiliations:** 1 Center for Biomedical Engineering and Technology, University of Maryland School of Medicine, Baltimore, Maryland, 21201 United States of America; 2 Department of Anatomy and Neurobiology, University of Maryland School of Medicine, Baltimore, Maryland, United States of America

## Abstract

Prion or PrP^Sc^ is a proteinaceous infectious agent that consists of a misfolded and aggregated form of a sialoglycoprotein called prion protein or PrP^C^. PrP^C^ has two sialylated N-linked carbohydrates. In PrP^Sc^, the glycans are directed outward, with the terminal sialic acid residues creating a negative charge on the surface of prion particles. The current study proposes a new hypothesis that electrostatic repulsion between sialic residues creates structural constraints that control prion replication and PrP^Sc^ glycoform ratio. In support of this hypothesis, here we show that diglycosylated PrP^C^ molecules that have more sialic groups per molecule than monoglycosylated PrP^C^ were preferentially excluded from conversion. However, when partially desialylated PrP^C^ was used as a substrate, recruitment of three glycoforms into PrP^Sc^ was found to be proportional to their respective populations in the substrate. In addition, hypersialylated molecules were also excluded from conversion in the strains with the strongest structural constraints, a strategy that helped reduce electrostatic repulsion. Moreover, as predicted by the hypothesis, partial desialylation of PrP^C^ significantly increased the replication rate. This study illustrates that sialylation of N-linked glycans creates a prion replication barrier that controls replication rate and glycoform ratios and has broad implications.

Prion diseases are a family of lethal, transmissible, neurodegenerative disorders that can be sporadic, inheritable or transmissible in origin^1^,^2^. A central event underlying the molecular mechanism of prion diseases involves conformational changes of the cellular form of the prion protein (PrP^C^) into the disease-associated transmissible form (PrP^Sc^)^1^. Multiple alternative, conformationally distinct, self-replicating states referred to as prion strains that give rise to different prion disease phenotypes can be produced within the same host or PrP^C^ amino acid sequence^3^,^4^,^5^,^6^. Among the parameters identified over the years that control prion transmission and replication are the amino acid sequence of PrP^C^ and strain-specific structure of PrP^Sc^^7^. PrP^C^ is subject to two types of posttranslational modifications, which include attachment of GPI anchor to the C-terminal residue Ser-231 and up to two N-linked carbohydrates to residues Asn-181 and Asn-197^8^,^9^,^10^,^11^. Roles of N-linked glycans and the GPI-anchor in prion transmission, spread, and pathogenesis have been the subjects of a number of studies in the last decade^12^,^13^,^14^,^15^,^16^.

The N-linked glycans are sialylated at the terminal positions, with each glycan being able to carry up to five sialic acid residues^17^. Despite the discovery of PrP sialylation more than 25 years ago, the potential role of sialylation in prion replication has not been explored. Terminal sialylation of glycans significantly alters the physical properties of both states, PrP^C^ and PrP^Sc^. In the absence of posttranslational modifications, the theoretical pI of the full-length prion protein is 9.6 and the estimated charge at pH 7.5 is + 9.5^18^. However, due to glycan sialylation, the actual pI of PrP molecules was found to be highly heterogeneous and spread from pH 9.6 to acidic pH^18^,^19^. In intact PrP^Sc^, the glycans are believed to be directed outwards, with the terminal sialic acid residues located at the interface with the extracellular environment or solvent^20^,^21^,^22^. Because sialic acid residues create a dense negative charge on the PrP^Sc^ surface, glycan sialylation might play a major role in modulating interactions between PrP^Sc^ and PrP^C^ and defining prion replication rate.

In previous study, we showed that sialylation level of PrP^Sc^ controls prion infectivity^18^. We also noticed that changes in sialylation levels of PrP^C^ influence PrP^Sc^ replication rate and the ratio of three glycoforms within newly formed PrP^Sc^. These findings led to a new hypothesis that electrostatic repulsion between sialic acid residues creates a replication barrier that slows down prion replication. Moreover, as diglycosylated glycoforms have more sialic acid residues per PrP molecule than mono- or unglycosylated glycoforms, this hypothesis also proposes that sialylation controls PrP^Sc^ glycoform ratios. In previous studies that examined prions formed in familial and sporadic prion diseases, the three glycoforms were found to be recruited into PrP^Sc^ in a selective fashion^23^. PrP^Sc^ glycoform ratios are believed to be an intrinsic characteristic of prion strains or subtypes and, as such, have been used for strain typing^24^,^25^. However, it still remains puzzling as to why the proportions of three glycoforms vary in a strain-specific manner, even within the same host. Here we propose that diglycosylated glycoforms are selectively excluded to reduce electrostatic repulsion between sialic acid residues within PrP^Sc^. The current study tests the above hypothesis on the role of sialylation in controlling the prion replication barrier and glycoform ratios.

## Results

### Sialylation status controls strain-specific glycoform ratio of PrP^Sc^

**Figure 1:**
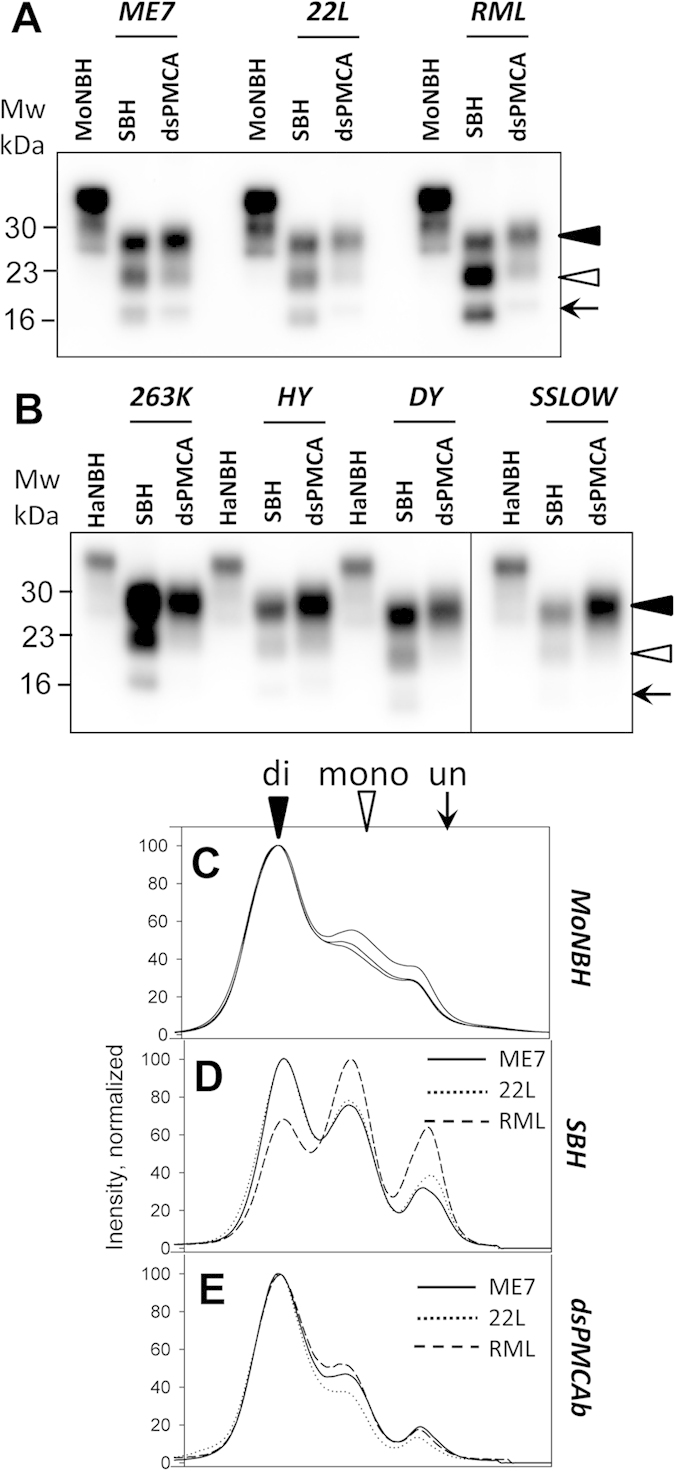
Analysis of glycoform profiles of brain- and dsPMCAb-derived materials. Western blots of scrapie brain-derived (SBH) and dsPMCAb-derived materials of mouse strains ME7, 22L and RML (**A**) or hamster strains 263K, HY, DY, SSLOW (**B**). For producing dsPMCA-derived material, dsPMCAb reactions were seeded with brain material and propagated in serial dsPMCA to the final dilution of brain material 10^8^-fold. Mouse or hamster normal brain homogenates (MoNBH or HaNBH, respectively) are shown as references. All samples except MoNBH and HaNBH were treated with PK. Mouse and hamster materials were stained with Ab3531 or 3F4 antibodies, respectively. Black triangles, white triangles and arrows mark di-, mono- and non-glycosylated glycoforms, respectively. Glycoform profiles of mouse PrP^C^ from three independent normal brain materials (**C**), scrapie brain-derived material (**D**) or dsPMCAb-derived material (**E**) for ME7 (solid lines), 22L (dotted lines) and RML (dashed lines). Profiles were built and normalized as described in Materials and Methods using results of 1D Western blots.

For establishing a relationship between sialylation status and glycoform ratios, a panel of hamster (263K, HY, DY and SSLOW) and mouse (ME7, 22L and RML) strains were employed. To produce PrP^Sc^ with reduced sialylation levels, normal brain homogenate (NBH) was treated with sialidase from *Arthrobacter ureafaciens*, which has broad substrate specificity and cleaves α2–3 and α2–6 linked sialic acid residues on N-linked glycans. Sialidase treated NBH was used as a substrate for PMCAb (a PMCAb format that utilizes desialylated substrate will be referred to as dsPMCAb) as previously described^18^. Western blots were used to compare the glycoform ratios in PrP^Sc^ and dsPMCAb products (Fig. 1). All mouse and hamster strains tested showed significant increase in diglycosylated glycoforms at the expense of mono- and unglycosylated glycoforms in dsPMCAb products in comparison to corresponding brain-derived materials (Fig. 1A,B). All three mouse strains (ME7, 22L, RML) showed considerable amounts of mono- and unglycosylated glycoforms in dsPMCAb products relative to that of dsPMCAb-derived material of hamster strains (263K, HY, DY, SSLOW). This higher proportion of mono- and non-glycosylated forms is a distinct trait of mouse strains, as all brain-derived mouse materials also showed higher proportions of mono- and unglycosylated forms relative to those of hamster strains^26^. Nevertheless, a considerable shift in glycoform ratios in favor of diglycosylated forms was observed in all mouse and hamster strains.

**Figure 2:**
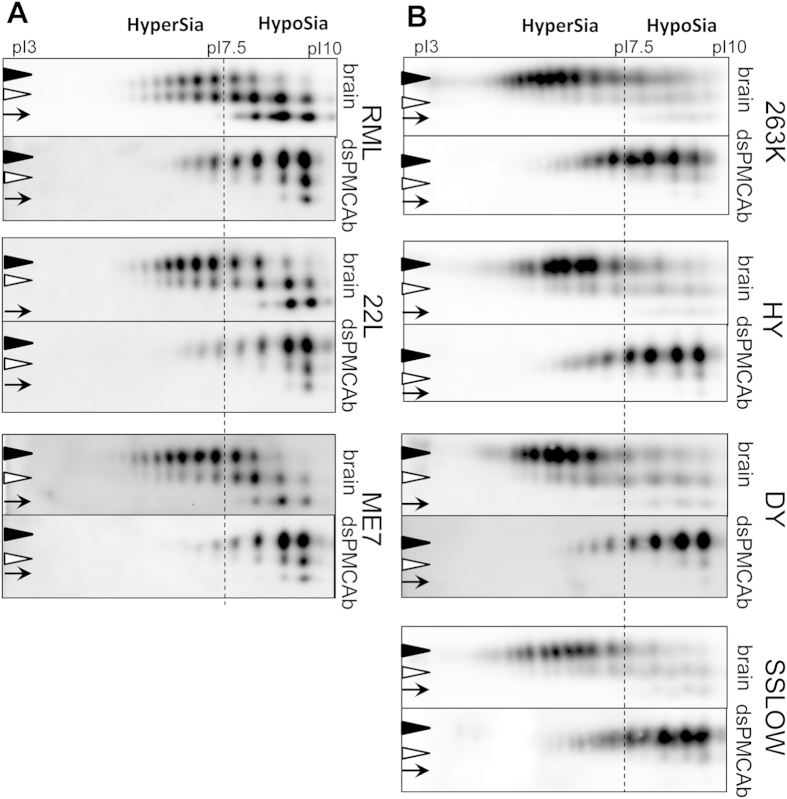
Analysis of sialylation status of scrapie brain- and dsPMCAb-derived materials. 2D analysis of charge distribution of scrapie brain- and dsPMCAb-derived materials for mouse strains RML, 22L and ME7 (**A**) and hamster strains 263K, HY, DY and SSLOW (**B**). dsPMCAb-derived materials were produced as described in the legend to Fig. 1. The dash line shows position of pI 7.5 and arbitrary divides “hypersialylated” and “hyposialylated” PrP molecules. Appearance of more than one charge isoform for unglycosylated PrP is attributed to structural heterogeneity of the GPI anchor^8^. Nevertheless, due to sialylation of glycans, monoglycosylated PrPs can carry up to five negatively charged sialic acid residues, while diglycosylated PrPs – up to ten sialic acid residues. As a result, the distribution of charge isoform is shifted toward acidic pH for monoglycosylated PrPs and even more so for diglycosylated PrPs, relative to that of unglycosylated PrPs. All samples were treated with PK. Mouse and hamster materials were stained with Ab3531 or 3F4 antibodies, respectively. Black triangles, white triangles and arrows mark di-, mono- and non-glycosylated glycoforms, respectively.

The differences in sialylation status between brain- and dsPMCAb-derived materials were analyzed using 2D gel electrophoresis. The two N-linked glycans can carry up to five terminal sialic acid residues each adding negative charges to individual PrP molecules^17^. Because the samples were denatured prior to 2D gel electrophoresis, the distribution of charge isoforms in the horizontal dimension reflects sialylation status of individual PrP molecules, where hypersialylated molecules run toward acidic pH and hyposialylated toward basic pH^18^. Consistent with previous studies^27^,^28^, unglycosylated forms that lack glycans and glycan-linked sialylation typically showed more than one charge isoforms (Fig. 2). This charge heterogeneity is attributed to the structural heterogeneity of the GPI anchors, a fraction of which could be also subject to single sialylation^8^. Unglycosylated forms could serve as a reference for charge heterogeneity resolved by 2D not attributable to sialylation of N-linked glycans. For all strains tested, the distribution of dsPMCAb-derived products was shifted towards basic pH relative to that of brain-derived materials as expected (Fig. 2). Such shifts document changes in sialylation status of dsPMCAb-derived materials that were produced from a partially desialylated substrate.

**Figure 3:**
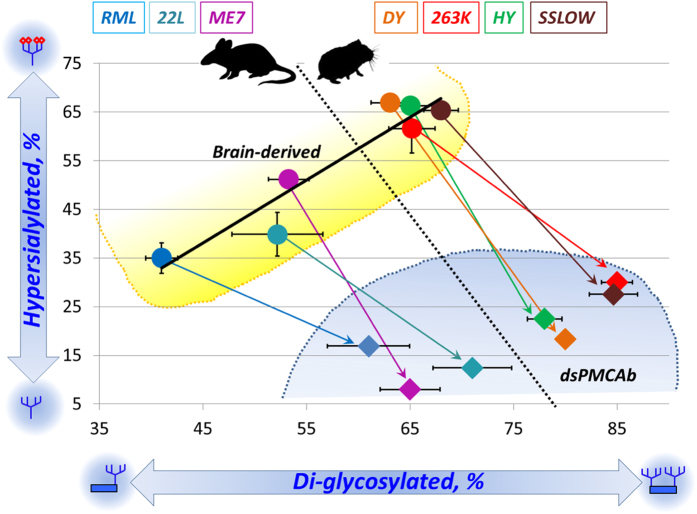
Correlation between PrP^Sc^ sialylation status and glycoform ratio. Percentage of diglycosylated glycoforms plotted as a function of percentage of hypersialylated isoforms for brain-derived (circles) and dsPMCAb-derived materials (diamonds) for mouse (RML, 22L and ME7) and hamster (263K, HY, DY and SSLOW) strains. Arrows show shifts in glycoform ratio after amplification in dsPMCAb. Percentage of diglycosylated glycoforms and hypersialylated isoforms are calculated as described in Materials and Methods. For each strain, at least three glycoform ratio values were acquired from independent brain materials or dsPMCA reactions; the variations were used to calculate mean and standard deviations. For RML, 22L and 263K brain materials at least three sialylation ratio values were available, permitting to calculate mean and standard deviations. Black solid line shows the result of linear fitting of the percent of diglycosylated as a function of the percent of hypersialylated for brain-derived PrP^Sc^, R^2^ = 0.89.

To analyze individual sialylation profiles, charged isoforms were separated arbitrarily into two categories, as described in Methods. Isoforms located toward acidic pH from pI 7.5 will be designated as hypersialylated and those toward basic pH will be designated as hyposialylated. The percentage of sum intensities of hypersialylated isoforms relative to the total intensities of all isoforms was used to compare the samples. To establish a correlation between sialylation status and glycoform ratio, the percentage of PrP^Sc^ diglycosylated forms was plotted as a function of the percentage of hypersialylated isoforms for brain- and dsPMCAb-derived materials (Fig. 3). This plot revealed a relationship between sialylation status and glycoform ratio: a decrease in sialylation levels of dsPMCA-derived products always resulted in considerable increase in recruitment of diglycosylated forms into PrP^Sc^ at the expense of mono- and unglycosylated glycoforms. This correlation was observed for all strains tested from two different hosts.

In summary, the changes in glycoform ratios in response to changes in sialylation status support the hypothesis that negative charges of sialic acid residues create electrostatic repulsion that limits recruitments of diglycosylated glycoforms. Partial desialylation of PrP^C^ abolishes electrostatic repulsion, and as a result, the proportion of diglycosylated glycoforms rises. The following testable predictions were postulated based on the above hypothesis. First, it is predicted that negative selection of diglycosylated forms upon conversion of PrP^C^ into PrP^Sc^ takes place, i.e the proportion of diglycosylated glycoforms in PrP^Sc^ should be lower than that in PrP^C^. The extent of negative selection should depend on a strain-specific structure and degree of electrostatic repulsion. For strain structures with close proximity of glycan moieties, the negative selection should be the strongest. Second, to counteract electrostatic repulsion, it is predicted that negative selection of hypersialylated diglycosylated isoforms takes place in addition to negative selection of diglycosylated forms in general. This negative selection should be the strongest for those strains that show the strongest negative selection of diglycosylated glycoforms, i.e. for strains with the highest proportion of monoglycosylated forms. Third, if glycan sialylation indeed creates a barrier for prion conversion, desialylation of PrP^C^ is predicted to remove this barrier and lead to an increase in prion amplification rates.

### Negative selection of diglycosylated forms upon conversion of PrP^C^ into PrP^Sc^

To test whether diglycosylated forms are underrepresented in PrP^Sc^ in comparison to that of PrP^C^, glycoform profiles of three mouse strains were compared to that of mouse PrP^C^ (Fig. 1A,C–E). Consistent with previous studies^26^, in PrP^C^ the diglycosylated forms were found to be predominant, while unglycosylated were the least represented (Fig. 1C). In PrP^Sc^ of all three strains, the proportion of di- versus mono- and unglycosylated forms shifted in favor of mono- and unglycosylated glycoforms (Fig. 1A,B). This result argues that the diglycosylated form is subject to negative selection upon conversion of PrP^C^ into PrP^Sc^. The shift was most substantial for the RML strain, illustrating that the extent of a negative selection is determined by a strain-specific structure. Remarkably, upon desialylation of the substrate, the dsPMCA-derived products showed a glycoform profile reminiscent of that of normal substrate, where diglycosylated forms were predominant (Fig. 1C,E). Moreover, in dsPMCA-derived products all three strains exhibited a very similar glycoform profile (Fig. 1E). These results demonstrate that reducing the content of sialic acid residues on PrP^C^ glycans result in recruitment of the three glycoforms into PrP^Sc^ proportionally to their relative populations in a substrate. A similar trend was found in hamster strains, although it was less pronounced (Fig. 1B), because diglycosylated forms were predominant in brain-derived PrP^Sc^. Together, these results illustrate that diglycosylated glycoforms are underrepresented in PrP^Sc^, the extent of this effect is strain- and species-specific, and that PrP^Sc^ structures of hamster strains accommodate diglycosylated glycoforms better than those of mouse strains.

### Negative selection of hypersialylated diglycosylated isoforms

**Figure 4:**
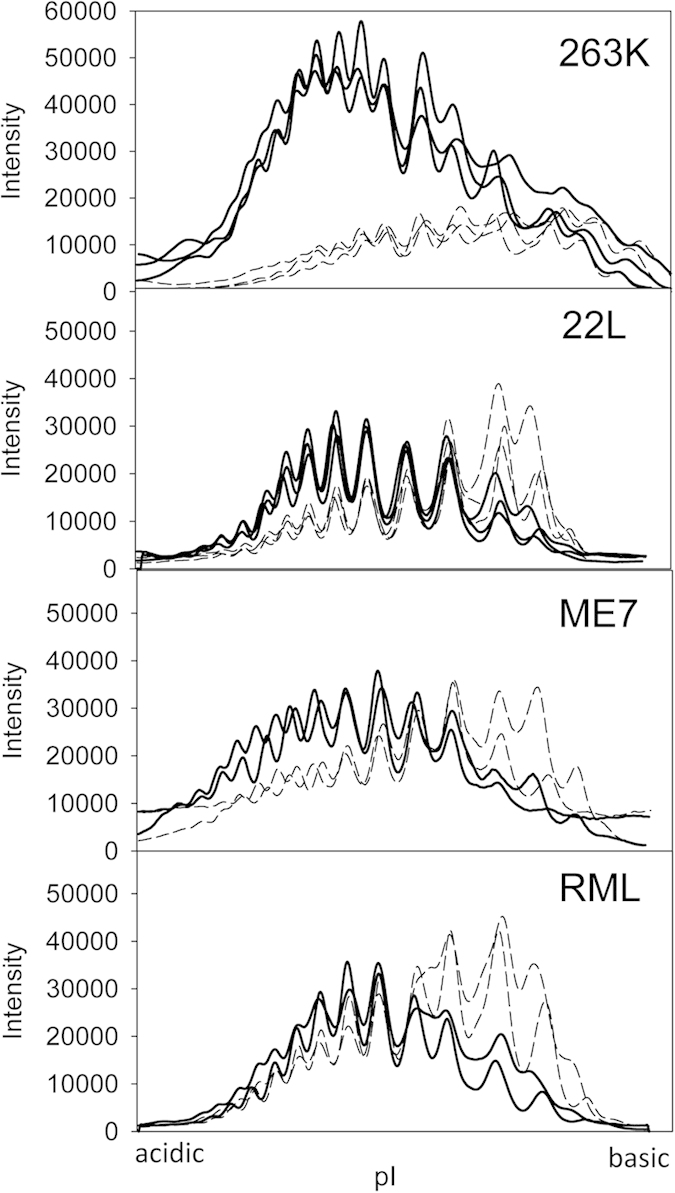
Sialylation profiles of di- and mono-glycosylated glycoforms of brain-derived materials. Sialylation profile of di- (solid lines) or mono-glycosylated (dashed lines) glycofoms of brain-derived materials for 263K, 22L, ME7 and RML. Profiles were built as described in Materials and Methods using results of 2D Western blots.

Among three glycoforms, diglycosylated PrP exhibit the highest charge heterogeneity, as it can accommodate up to ten negatively charged sialic acid residues on two glycans per molecule^17^. As a result, the charge distribution of diglycosylated isoforms is shifted toward acidic pH and typically extends beyond that of monoglycosylated isoforms, which accommodate only up to five sialic residues per molecule. The extent to which diglycosylated isoforms migrate beyond monoglycosylated isoforms toward acidic pH reports on recruitment of hypersialylated diglycosylated isoforms. Careful analysis of 2D results revealed that in brain-derived PrP^Sc^ hypersialylated diglycosylated molecules were recruited in a strain-specific manner (Fig. 2). Hamster strains showed the most substantial extension of diglycosylated isoforms toward acidic pH relative to the distribution of monoglycosylated isoforms (Figs 2B and 4). This observation suggests that hypersialylated diglycosylated molecules are well represented in PrP^Sc^. It is also consistent with the previous results showing that structures of hamster strains accommodate diglycosylated PrP relatively well. In contrast to hamster strains, in mouse strain RML the distribution of diglycosylated isoforms does not extend beyond that of monoglycosylated isoforms at acidic pH (Figs 2A and 4). This indicates that a negative selection of hypersialylated diglycosylated molecules takes place in addition to a general negative selection of diglycosylated glycoforms. For the other two mouse strains 22L and ME7, the diglycosylated isoforms extended beyond monoglycosylated isoforms at acidic pH, although not to such a degree as those of hamster strains (Figs 2A and 4). Overall, the strain-specific structures that tolerate less diglycosylated forms also tend to exclude hypersialylated diglycosylated forms. In fact, plotting strain-specific percentage of diglycosylated isoforms versus percentage of hypersialylated isoforms revealed a correlation between the two parameters that fits well to a linear function with R-squared value of 0.89 (Fig. 3).

### Sialylation of PrP^C^ glycans controls replication barrier

**Figure 5:**
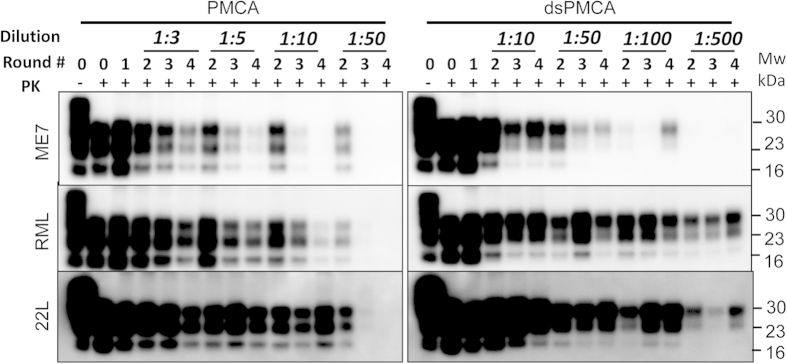
Analysis of amplification rates in PMCAb and dsPMCAb. Mouse scrapie brain materials were diluted 10^2^-fold into 10% МоNBH or desialylated МоNBH and subjected to four serial PMCAb or dsPMCAb rounds. The material amplified in each round was diluted to a specified dilution fold for the next round as indicated. Unamplified scrapie seeds diluted into NBH are shown as round 0. Samples were treated with PK as indicated. All blots were stained with Аb3531 antibody.

It is predicted that sialylation of PrP^C^ glycans creates a replication barrier. To test this hypothesis, we compared amplification rates of ME7, RML and 22L in normal versus desialylated substrates using PMCAb and dsPMCAb, respectively. For comparing amplification rates, sets of serial PMCAb or dsPMCAb reactions were conducted with dilution folds between serial rounds starting from 1:3 and higher (Fig. 5). The amplification rate is defined operationally as the highest dilution between serial rounds at which amplification is still capable of compensating the effect of dilution as described previously^29^,^30^. In desialylated substrate, the amplification rate increased from 5- to 100-fold for ME7 and from 10- to at least 500-fold for RML and 22 L. Notably, consistent with previous results, dsPMCAb-derived products showed a higher proportion of diglycosylated glycoforms relative to PMCAb-derived products in all three strains (Fig. 5).

## Discussion

**Figure 6:**
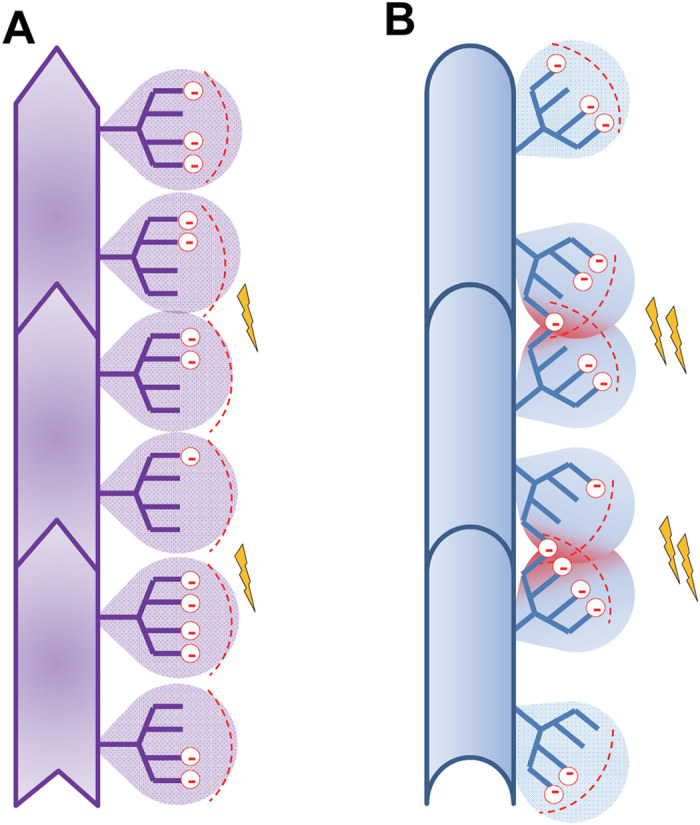
Schematic diagram illustrating how relative position of two glycans creates structural constraints. (**A**) A strain with minor clashes between glycans that belong to neighboring PrP monomers can recruit diglycosylated monomers. This strain is characterized by low electrostatic repulsion between sialylated glycans (schematically marked by lightning bolts). (**B**) A strain with substantial spatial constraints between glycans from neighboring monomers limits percentage of diglycosylated molecules due to high electrostatic repulsion between sialic acids (red circles with negative charge) from neighboring N-glycans.

In the absence of posttranslational modifications, prion protein has a strong positive charge at physiological pH with the theoretical pI of 9.6^18^. Sialylation of glycans can add up to ten negatively charged residues and shifts the pI toward acidic pH, making the population of PrP molecules highly heterogeneous with respect to pI and the net charges at physiological pH^17^,^18^. In PrP^Sc^ particles, glycans are believed to be directed outward where the terminal sialic acid residues create a dense negative charge on the PrP^Sc^ surface^20^,^21^,^22^. The current study proposed that due to electrostatic repulsion, sialylation of glycans creates structural constraints for PrP^Sc^ replication. Several testable predictions were postulated based on this hypothesis. First, to reduce electrostatic repulsion, it is expected that PrP^C^ with a heavy sialylation level is partially excluded from the conversion. This could be achieved via (i) selective recruitment of mono- and unglycosylated PrP^C^ at the expense of diglycosylated PrP^C^ and (ii) preferential exclusion of hypersialylated diglycosylated PrP^C^. Depending on the strain-specific alignment of glycan moieties, the extent of this effect is expected to be strain-specific (Fig. 6). Second, an increase in the rate of prion amplification is expected when the constraints imposed by sialic acid residues are relieved by employing a substrate with low sialylation levels. In other words, sialylation of PrP^C^ glycans creates a replication barrier that can be reduced by reducing sialylation levels of a substrate.

In support of the above hypothesis, the current work showed that the relative population of diglycosylated forms was lower in PrP^Sc^ than those in PrP^C^, and that the negative selection of diglycosylated forms was strain-specific. RML showed the strongest exclusion of diglycosylated forms. The hamster strains (263K, HY, DY, SSLOW) had the weakest negative selection, while 22L and ME7 strains were in the middle. Remarkably, in PrP^Sc^ generated from PrP^C^ that was partially desialylated, the relative populations of glycofoms were found to be very similar to those of the substrate. This observation illustrates that the pressure for preferential selection of un- and monoglycosylated forms was released by cleaving sialic acid residues. In addition to the negative selection of diglycosylated forms, strains with the strongest structural constraints also excluded hypersialylated molecules. RML showed the strongest negative selection of diglycosylated and also hypersialylated forms. In fact, a strong correlation between strain-specific percentage of diglycosylated forms and hypersialylated forms presented in Fig. 3 for brain-derived material suggests that the negative selection of both is attributed to the same structural constrains.

The glycoform ratios within PrP^Sc^ are believed to be one of the intrinsic characteristics of prion strains or PrP^Sc^ subtypes (reviewed in^31^). In fact, the glycoform ratios have been used for strain typing and classification of CJD type^24^,^25^. It was not clear why three glycoforms are recruited by prion strains in different proportions even within the same host. The current work demonstrates that the strain-specific ratio of the three glycoforms is a result of negative selection of heavily sialylated PrP molecules. The extent to which heavily sialylated PrP is excluded is presumably controlled by the strain-specific structure of PrP^Sc^ and relative position of the two glycans (Fig. 6). Remarkably, when exposed to desialylated substrate, prion strains lose strain-specific selectivity toward different glycoforms and the glycoform ratio within PrP^Sc^ mirrors that of PrP^C^.

Brain-derived PrP^Sc^ of different strains was found to be different with respect to its sialylation status. (Fig. 3). In combination with substantial strain-specific differences in glycoform ratios, these differences are likely to produce distinct strain-specific density of sialic acid residues on a surface of PrP^Sc^ particles and distinct strain-specific pIs of PrP^Sc^. For instance, at physiological pH, RML PrP^Sc^ particles are expected to have net positive charges, whereas PrP^Sc^ particles of hamster strains are expected to be charged negatively (Fig. 2). Terminal sialic acid residues constitute key elements of diverse functional epitopes that are recognized by dozens of cell receptors including siglecs and selectins^32^,^33^. Through these functional epitopes, sialylation of glycoproteins mediate their interaction with cells of the innate immune system and microglia^34^. These interactions define the ultimate fate of mammalian cells, glycoproteins in circulation and microbial pathogens^35^,^36^. It remains to be established whether strain-specific differences in PrP^Sc^ sialylation play any role in defining strain-specific neurotropism and lymphotropism.

The current study illustrates that PrP^Sc^ glycoform ratio is not only controlled by strain or host, but also by sialylation level of PrP^C^. As such, experiments on infecting new hosts or cell lines with unknown PrP^C^ sialylation status should be interpreted with great care. In previous studies, shifts in glycoform ratios toward diglycosylated glycoforms were observed in scrapie-infected cells treated with swainsonine, a compound that inhibits complex glycosylation^37^,^38^. It was concluded that selection of minor strain variants or structural mutants occurred^37^,^38^. Because treatment with swainsonine blocks complex glycosylation including sialylation, it is expected that the glycoform ratios would change in favor of diglycosylated forms regardless whether selection of a new variants occurs or not. The current work predicts that if the PrP^Sc^ glycoform ratio changes upon infecting a new host, this change is indicative of an altered sialylation status of PrP^C^ and, specifically, a shift toward diglycosylated forms would suggest a reduced sialylation level of PrP^C^.

The hypothesis presented in the current work introduces new constraints for establishing PrP^Sc^ structure. To account for the negative selection of diglycosylated/hypersialylated PrP^C^, glycans of the neighboring PrP molecules should be aligned in close proximity to each other within assembled PrP^Sc^ particles. The average volume and dimension of glycan moieties should be taken into consideration for estimating the proper folding pattern and the number of intramolecular rings or layers acquired by a single polypeptide chain within intact PrP^Sc^. More substantial clashes between glycans of neighboring diglycosylated forms are expected for strains or PrP^Sc^ subtypes with predominantly monoglycosylated forms, such as sporadic CJD, in comparison with the strains where diglycosylated forms are predominant, such as new variant CJD^24^ (Fig. 6).

For the three mouse strains tested in the current study, partial desialylation of PrP^C^ increased the replication rates by 20–50 fold (Fig. 5). These results suggested that sialylation of PrP glycans contributes to a replication barrier. An alternative explanation is that sialidase treatment of NBH had an effect other than on PrP^C^. While this seems to be unlikely, this possibility should not be discarded completely. In previous studies, partial desialylation of PrP^C^ was also found to considerably reduce the barrier in cross-seeded PMCAb reactions^18^. Together, these data suggest that the replication barrier attributable to glycan sialylation is universal, i.e. it might not only control the rate of prion replication within the same host but also the barrier associated with prion transmission between different species. There might be several components that underlie a replication barrier attributable to sialylation. First, electrostatic repulsion between negatively charged sialic acid residues on glycans of PrP^Sc^ and PrP^C^ limits the range of PrP^C^ molecules suitable for conversion to those with moderate sialylation levels. Second, even for the PrP^C^ molecules that are suitable for conversion, electrostatic repulsion of surface glycans might slow down the rates of PrP^C^ binding to PrP^Sc^ and PrP^C^ conversion. Third, electrostatic repulsion between surface glycans of nascent PrP^Sc^ has a negative impact on thermodynamic stability of PrP^Sc^ particles, the effect that has to be counteracted by other forces that stabilize packing of polypeptide chains within PrP^Sc^ particles. Notably, while thermodynamic stability of PrP^Sc^ varies depending on strain-specific structure^6^,^29^,^39^,^40^, the range of strain-specific thermodynamic stabilities of PrP^Sc^ is typically lower than those of amyloid fibrils generated *in vitro* using recombinant PrP^41^,^42^. In part, this could be due to electrostatic repulsion between sialylated glycans that recombinant PrP lacks.

According to the hypothesis introduced here, the same structural constraints account for at least part of the replication barrier and the degree to which diglycosylated forms are excluded. If this is true, there should be a correlation between strain-specific glycoform ratios and the efficiency of prion replication. Indeed, mouse strains have a high proportion of monoglycosylated glycoforms and also slow replication rates in PMCA. Similar correlations between glycoform ratio and the replication efficiency was observed for two forms of CJD. It is known that nvCJD, which is predominantly diglycosylated, replicates in PMCA with a much better efficiency than sCJD, which is predominantly monoglycosylated^43^,^44^,^45^. However, not all strains with predominantly diglycosylated glycoforms display fast replication rates suggesting that factors other than sialylation contribute to the replication barrier. For instance, hamster-adapted strains DY, 139H and 22AH are predominantly diglycosylated, but replicate very slowly^39^.

## Methods

### Normal and scrapie brain materials

Weanling Golden Syrian hamsters or C57BL/6NHsd mice were inoculated intracerebrally under 2% O2/4 MAC isoflurane anesthesia using scrapie brain homogenates of hamster- or mouse-adapted strains, respectively. For the synthetic strain SSLOW, animals of fourth serial SSLOW passage were used for analysis^46^. Animals were euthanized at the terminal stage of the disease and brains were collected for subsequent analysis. This study was carried out in strict accordance with the recommendations in the Guide for the Care and Use of Laboratory Animals of the National Institutes of Health. The animal protocol was approved by the Institutional Animal Care and Use Committee of the University of Maryland, Baltimore (Assurance Number A32000-01; Permit Number: 0215002).

### Protein misfolding cyclic amplification with beads (PMCAb) and PMCAb in desialylated substrate (dsPMCAb)

10% normal brain homogenate (NBH) from healthy hamsters or mice was prepared as described previously^47^. To produce desialylated substrates for dsPMCAb, 10% NBH was treated with *Arthrobacter ureafaciens* sialidase (cat # P0722L, New England Biolabs, Ipswich, MA) as follows. After preclearance of NBH at 500 × *g* for 2 min and addition of the enzyme buffer supplied with the manufacturer to supernatant 200 units/mL of sialidase were added to the supernatant, incubated on a rotator at 37 °C for 5 h and the resulting material referred to as dsNBH substrate used in dsPMCAb.

PMCAb and dsPMCAb reactions were conducted as previously described^18^,^48^ using Misonix S-4000 microplate horn (Qsonica LLC, Newtown, CT) in the presence of two 2/32” Teflon beads in each tube (McMaster-Carr, Elmhurst, IL). One round consisted of 20 sec sonications delivered at 170 W energy output applied every 20 min during a 24 hour period. For each subsequent round, 10 μl (for hamster strains) or 20 μl (for mouse strains) of the reaction products from the previous round were mixed with 90 or 80 μl of fresh substrate, respectively, or as specified for the amplification rate experiment.

### Western blots

10 μL of 10% brain homogenate or dsPMCAb-derived materials were supplemented with 5 μL 1% SDS and 5 μL proteinase K (cat. # P8107S, New England Biolabs, Ipswich, MA), to a final concentration of 0.25% SDS and 25 μg/mL proteinase K, and incubated at 37 °C for 1 hour. Then, SDS sample buffer was added, samples were boiled for 10 minutes and loaded onto NuPage 12% BisTris gel (cat. # NP0341BOX, Life Technologies, Carlsbad, CA), transferred to PVDF membrane, and probed with 3F4 (cat. #SIG-39600, Covance) or Ab3531 antibody (Abcam).

### Preparation of brain- and dsPMCAb-derived materials for 2D

10% (wt/vol) of scrapie brain homogenates were prepared in PBS using glass/Teflon homogenizers attached to a cordless 12 V compact drill (Ryobi) as previously described^46^. For 2D electrophoresis of brain-derived materials, an aliquot of 10% (wt/vol) homogenate was diluted with nine volumes of 1% (vol/vol) Triton X-100 in PBS, sonicated for 30 s inside a Misonix S-4000 microplate horn (Qsonica LLC, Newtown, CT), and treated with 25 μg/mL proteinase K for 30 min at 37 °C. dsPMCAb materials were treated with 25 μg/mL PK in the presence of 0.25% SDS for 60 min at 37 °C. Resulting brain and dsPMCAb samples were supplemented with 4xSDS loading buffer, heated for 10 min in a boiling water bath and processed for 2D electrophoresis as described below.

### 2D electrophoresis

Samples of 25 μL volume prepared in loading buffer as described above were solubilized for 1 h at room temperature in 200 μL solubilization buffer (8 M Urea, 2% (wt/vol) CHAPS, 5 mM TBP, 20 mM TrisHCl pH 8.0), then alkylated by adding 7 μL of 0.5 M iodoacetamide and incubated for 1 h at room temperature. Then, 1150 μL of ice-cold methanol was added and samples were incubated for 2 h at −20 °C. After centrifugation at 16,000 g at 4 °C, supernatant was discarded and the pellet was re-solubilized in 160 μL rehydration buffer (7 M urea, 2 M thiourea, 1% (wt/vol) DTT, 1% (wt/vol) CHAPS, 1% (wt/vol) Triton X-100, 1% (vol/vol) ampholyte, trace amount of Bromophenol Blue). Fixed immobilized pre-cast IPG strips with a non-linear pH gradient 3–10 used in the previous study^18^ were replaced by fixed immobilized pre-cast IPG strips with a linear pH gradient 3–10 (cat. # ZM0018, Life Technologies, Carlsbad, CA) that provide a better resolution of charge isoforms. Strips were rehydrated in 155 μL of the resulting mixture overnight at room temperature inside IPG Runner cassettes (cat. # ZM0003, Life Technologies). Isoelectrofocusing (first dimension separation) was performed at room temperature with rising voltage (175V for 15 minutes, then 175–2,000V linear gradient for 45 minutes, then 2,000 V for 30 minutes) on Life Technologies Zoom Dual Power Supply, using the XCell SureLock Mini-Cell Electrophoresis System (cat. # EI0001, Life Technologies). The IPG strips were then equilibrated for 15 minutes consecutively in (i) 6 M Urea, 20% (vol/vol) glycerol, 2% SDS, 375 mM Tris-HCl pH 8.8, 130 mM DTT and (ii) 6 M Urea, 20% (vol/vol) glycerol, 2% SDS, 375 mM Tris-HCl pH 8.8, 135 mM iodoacetamide, and loaded on 4–12% Bis-Tris ZOOM SDS-PAGE pre-cast gels (cat. # NP0330BOX, Life Technologies). For the second dimension, SDS-PAGE was performed for 1 h at 170 V. Immunoblotting was performed as described elsewhere, blots were stained using 3F4 or Ab3531 antibody.

### Western blot densitometry analysis

1D or 2D western blot signal intensity was digitized for densitometry analysis using AlphaView software (ProteinSimple, San Jose, CA). For densitometry analysis of the glycoform distribution (expressed as a percentage of the diglycosylated form), target bands were selected using a uniform rectangular sampling area that encompassed the band of interest. Background optical density of an equal area from the same blot was determined and subsequently subtracted from the density of the bands. At least three measurements from independent gel loadings were used for each sample for calculating mean and standard deviations.

For analysis of sialylation, 2D blots were aligned horizontally and a line drawn at pI 7.5 was used to arbitrarily separate charge isoforms into hypersialylated and hyposialylated. In the software window, a rectangle was drawn to confine the spots of interest, and the densities measured. The intensity of an equal background area from the same blot was subtracted before calculations were done. The acquired spot ensemble intensities were used to calculate percentage of “hypersialylated” isoforms. Hypersialylated isoforms were defined operationally as all isoforms located to the left of the pI7.5 line; the percentage of hypersialylated isoforms was calculated assuming the total intensity of all isoforms as 100%.

For generating glycoform profiles or sialylation profiles for di- and monoglycosylated glycoforms, densitometry analysis of 1D or 2D blots, respectively, was performed using the “Lane profile” function in the AlphaView program. For data presented in normalized format (Fig. 4), the highest curve signal value was taken as 100%.

## Additional Information

**How to cite this article**: Katorcha, E. *et al.* Sialylation of the prion protein glycans controls prion replication rate and glycoform ratio. *Sci. Rep.* **5**, 16912; doi: 10.1038/srep16912 (2015).
